# Anti-N-methyl-d-aspartate Receptor Encephalitis Related Sinus Node Dysfunction and the Lock-Step Phenomenon

**DOI:** 10.12691/ajmcr-8-12-20

**Published:** 2020-09-25

**Authors:** Krunal H. Patel, Yuvraj Chowdhury, Mrinali Shetty, Vaibhavi Uppin, Paul Madaj, Moro O. Salifu, Mary Youssef, Victoria L. Henglein, Samy I. McFaralne

**Affiliations:** 1 Department of Internal Medicine, State University of New York, Downstate Medical Center, Brooklyn, NY, USA - 11203; 2 Division of Cardiovascular Disease, State University of New York, Downstate Medical Center, Brooklyn, NY, USA - 11203; 3 Division of Cardiovascular Disease, University of Chicago (NorthShore), Evanston, IL, USA - 60201

**Keywords:** anti-N-methyl-d-aspartate receptor encephalitis (ANMDARE), Lock Step Phenomenon, autoimmune encephalitis, ovarian teratoma, autonomic dysregulation, sinus node dysfunction

## Abstract

Described in 2007, anti-N-methyl-d-aspartate receptor encephalitis (ANMDARE) is a rare autoimmune limbic encephalitis affecting young adults (predominantly women of reproductive age) and is a paraneoplastic manifestation of ovarian teratoma in about half of the cases. ANMDARE is characterized by psychiatric changes, neurological changes, autonomic instability and cardiac dysrhythmias. In this report, we present a 36-year-old woman who was 16 weeks pregnant and brought to the hospital with confusion and subsequently had a seizure with Electroencephalography (EEG) demonstrated an extreme delta brush pattern consistent with ANMDARE. Patient developed sinus nodal dysfunction and was also found to have ovarian teratoma, a rather typical presentation for ANMDARE, that is considered a paraneoplastic syndrome for ovarian teratoma. In this report, we highlight the cardiac manifestation of ANMDARE, the pathophysiology associated with autonomic instability, and management strategies of this rare, and largely devastating illness.

## 1. Introduction

ANMDARE is a rare and severe form of autoimmune encephalitis affecting primarily young adults and children at an incidence rate of about 5 to 8 per 100,000 [1]. Though only recently described, ANMDARE is the third most common form of encephalitis after viral encephalitis and acute disseminated encephalitis [1]. ANMDARE may present with a wide array of manifestations ranging from symptoms such as decreased consciousness, insomnia and defects in memory recall to more severe manifestations like seizures and psychiatric changes. Consequences of autonomic dysregulation are the primary cause of death [2]. Autonomic dysregulation can manifest as bradycardia, tachycardia, cardiac pauses, hypo/hyperthermia, blood pressure dysregulation and hypoventilation [3]. ANMDARE affects the brain primarily by attacking the extracellular NMDA receptors on neuronal surfaces which lead to internalization of the receptor and inflammation [4]. Internalization of the antigen-antibodies complex result in intracellular inflammation and neuronal destruction. Antibodies generated against the NMDA receptor may represent an underlying paraneoplastic process, most commonly ovarian teratomas [5]. The pathophysiology is a cross immune response secondary to molecular mimicry between the teratoma antigens and neuronal NMDA receptors [5]. Our case highlights the cardiac complications associated with ANMDARE followed by an in-depth review of the topic.

## 2. Case Presentation

**Figure 1.**
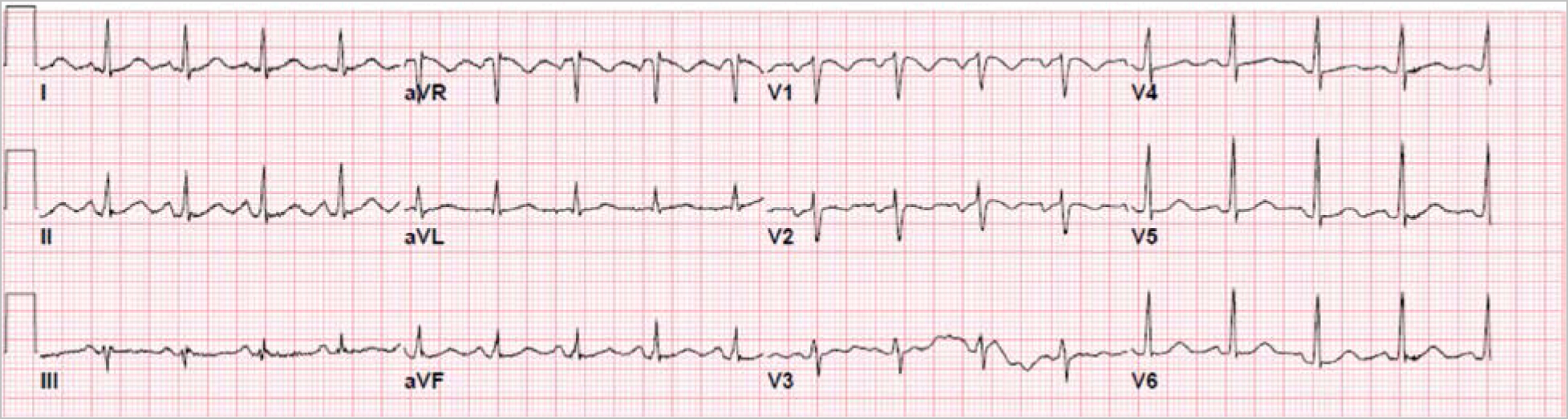
Sinus Tachycardia 110 bpm (Admission EKG)

A 36-year-old woman who was 16-weeks pregnant with no known past medical history was brought to the hospital by her sister for 2 weeks of headaches and insomnia, followed by vivid hallucinations and bizarre delusions. Her vital signs at arrival were significant for a heart rate of 104 bpm, temperature of 97.6^0^ F, blood pressure of 111/64 mmHg and a respiratory rate of 18 breaths per minute saturating of 100% on room air. She was agitated, disoriented and speaking incoherently. Her cardiac and respiratory exams were normal. Neurological evaluation was limited as the patient was agitated and non-cooperative. Her deep tendon reflexes were +2 in bilateral upper and lower extremities. She was moving all extremities and reacted to pain stimuli. EKG on admission was significant for sinus tachycardia at 110 bpm [Figure 1]. CT scan of the head which was negative for any intracranial bleeding or intracranial masses. Arterial blood gas analysis, thyroid function testing, blood cultures and urine analysis were noncontributory [Table 1].

On day 2 of admission, the patient experienced refractory generalized tonic-clonic seizures necessitating endotracheal intubation for airway protection. Evaluation with an MRI of the brain, MRA and MRV of the cerebral circulation did not reveal any intracranial pathology. Cerebrospinal fluid analysis showed lymphocytic pleocytosis [Table 1]. Electroencephalography (EEG) demonstrated an extreme delta brush pattern consistent with ANMDARE. ANMDARE antibody and glial fibrillary acidic protein antibody positivity confirmed the diagnosis of ANMDARE. During her seizure episodes the patient demonstrated rhythmic oral grimacing and intermittent adduction of bilateral lower extremities. These findings correlated with the delta brush pattern seen on EEG. The patient required four anti-epileptic agents (phenobarbital, levetiracetam, clobazam, and lacosamide) to suppress the seizures. NMDARE was subsequently treated with 5 cycles of plasmapheresis, a course of stress dose methylprednisolone*, IVIG and rituximab.

**Figure 3.**
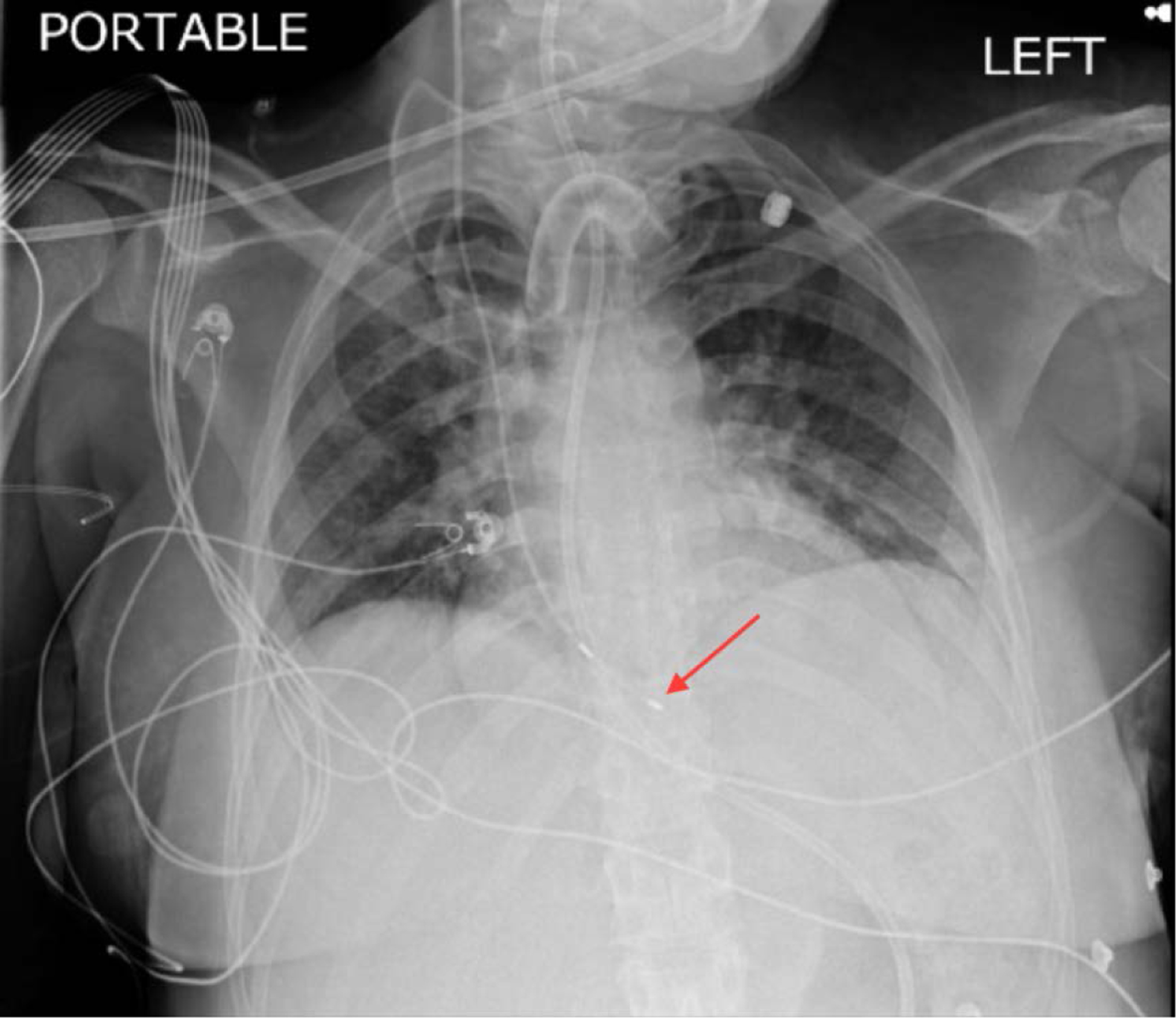
Red arrow showing successful placement of transvenous pacemaker tip

During the course of her stay, telemetry identified sinus node dysrhythmias. Episodes of sinus tachycardia alternating (HR ~ 110–125 bpm) [Figure 2A] with sinus bradycardia (HR ~ 30–35 bpm) [Figure 2B] and sinus arrest (longest pause ~ 7 secs) [Figure 2B] related and unrelated to vagal stimuli (ultrasound fetal monitoring). A transvenous pacemaker was placed through the right internal jugular vein [Figure 3].

Once ANMDARE was diagnosed, evaluation for a potential precipitating cause was undertaken. MRI of the abdomen demonstrated a left ovarian complex cystic structure measuring 32 × 33 mm. Given the association between teratomas and ANMDARE, bilateral oophorectomy was performed. Pathology identified hair and sebaceous secretions in the cyst and confirmed the diagnosis of ovarian teratoma. Despite treatment and oophorectomy patient currently remains dependent on the ventilator with poor neurological recovery at 32 weeks of gestation. Her sinus node dysrhythmia recovered in 8 days precluding the need for the placement of a permanent pacemaker.

## 3. Discussion

ANMDARE is a rare disorder which predominantly affects adults between the ages of 24 to 35 [6]. Women have a four times higher incidence than men. In women of reproductive age who are diagnosed with ANMDARE, 46% are eventually diagnosed with an underlying teratoma. The prevalence of bilateral teratoma in patients diagnosed ANMDARE is as high as 15% [7].

The key to diagnosing ANMDARE early is to have a high degree of clinical suspicion in a young patient with sudden onset of psychiatric changes, autonomic instability and seizures. Prognosis is largely dependent on early diagnosis and treatment. Treatment with high dose methylprednisolone, intravenous immunoglobulins (IVIG), and plasma exchange and early surgical removal of associated teratomas have been found to improve outcomes the most [8,9,10]. Patients refractory to treatment with the above modalities are given a trial of rituximab and cyclophosphamide. The largest published retrospective study of 501 patients with ANMDARE showed that nearly all (97%) patients that were treated with first-line immunotherapy, methylprednisolone, IVIG, and/or PLEX had symptoms improve within the first four weeks [10].

Our discussion focuses primarily on the sinus dysrhythmias associated with ANMDARE, and their management. Sinus bradycardia, sinus pause and sinus tachycardia have all been associated with ANMDARE and are attributed to severe autonomic dysregulation [11]. The largest retrospective study of 100 patients with ANMDARE, demonstrated that about two thirds of the patients went onto develop autonomic instability and about one third developed cardiac arrhythmias [26]. The dysrhythmias seen were tachycardia (53%), bradycardia (19%), or both (38%) [26,27]. The central autonomic control of the heart is an intricate and complex entity. Resting heart rate is a function of the cardiac pacemaker which has dual innervations: the parasympathetic system which is negatively chronotropic, and the sympathetic system which is positively chronotropic [12].

Brain mapping has identified multiple regions which are involved in cardiac pacemaker regulation. The telencephalon, anterior cingulate cortices, insula, and the amygdala all modulate the parasympathetic and sympathetic innervation of the heart [13,14]. The parasympathetic innervation of the heart is mediated through the nucleus ambiguous, located in the medulla oblongata, which via the vagus nerve, innervates the epicardial ganglionic plexus of the heart [15]. The sympathetic regulation is coordinated by the rostral ventrolateral medulla, also located in the medulla oblongata, which through excitatory inputs, innervates the intermediolateral cell column located in the thoracic spinal column [16]. These excitatory nerves then proceed to innervate the cardiac ganglia resulting in positive inotropic and chronotropic effects [17].

Three mechanisms of autonomic dysregulation that is seen in patients with ANMDARE have been postulated [18]. An animal study by Lathers *et al*, showed the correlation of epileptogenic activity and the associated changes in the autonomic cardiac function and subsequent development of arrhythmias [19]. The study simultaneously monitored EEGs, vagal parasympathetic discharges, and postganglionic sympathetic discharges during an epileptic episode. It was observed that during epileptiform activity there was simultaneous postganglionic cardiac sympathetic and vagal parasympathetic discharge. This phenomenon was termed the Lock-Step Phenomenon (LSP) [19,20]. LSP has been theorized as being causative of the fatal bradyarrhythmias, tachyarrhythmias and pauses seen with epileptiform activity in NMDARE [21].

Another proposed mechanism of dysrhythmias in ANMDARE, is the downregulation of NMDA receptors due to the binding of the anti-NMDA receptor antibodies (anti-NMDARA) [22]. Anti-NMDARA bind to the NMDA receptor and result in the internalization of the antibody-receptor complex. The receptor becomes unavailable for binding to extracellular NMDA and results in subsequent autonomic dysregulation [23]. Animal studies and autopsies have established that the anti-NMDARA mediated internalization of the NMDA receptor occurs primarily in the telencephalon, anterior cingulate cortices, insula and the amygdala [22,24]. NMDA receptor inactivation in these regions causes dysregulation of the vagal stimulation of the heart [24]. The downregulation of the NMDA receptors leads to the loss of cardiac parasympathetic stimulation and results in inappropriate sinus tachycardia. The loss of NMDA receptor mediated vagal tone then causes compensatory upregulation of secondary pathways that respond to afferent vagal stimuli leading to inappropriate bradycardia and sinus arrest. This leads to periods of sustained sinus tachycardia alternating with bradyarrhythmia [24]. Lastly, dysrhythmias in ANMDARE can manifest as Cushing’s triad (widened pulse pressures, bradycardia, and irregular respirations) due to the elevated intracranial pressure (ICP) [25].

The behavioral and psychiatric manifestations within this rare disease have been highlighted in both a book and a movie, called ‘Brain on Fire’, spreading more awareness of the unique presentation patients with ANMDARE often display and the social implications they may experience [28,29]. The book as well as the movie surround the case of a young female reporter, who presented with the classic manifestations of ANMDARE, experiencing seizures, high blood pressure, psychotic behavior, hallucinations, etc. and fit the characteristic clinical diagnostic criteria (young, female of reproductive age). However, she was initially diagnosed with psychiatric problems, suffering numerous additional symptoms from prescribed medications that did not address the underlying pathology. The diagnosis was finally made after a thorough examination was conducted by another physician who asked her to draw a clock on a piece of paper, in which she drew the numbers 1–12 completely on the right side of the clock, leaving the left side blank, indicating neuroinflammation in the right side of her brain responsible for left-side spatial neglect [28]. These media presentations of this specific case of ANMDARE illustrates a common problem in medicine today; symptoms and behaviors caused by neurological diseases can often be misdiagnosed and incorrectly treated for psychiatric diseases. While the producer of the movie used some dramatic effects necessary for artistic presentations, the book remains faithful in the clinical description of this disorder, highlighting the importance of understanding the pathophysiology, along with early diagnosis and treatment in ANMDARE, a potentially devastating disorder.

## 4. Conclusion

ANMDARE has been shown to cause sinus node dysfunction in the form of sinus arrest, sinus bradycardia and inappropriate sinus tachycardia in a large proportion of patients. Mechanisms proposed to explain the sinus arrhythmias are the lock-step phenomenon during epileptic activity, the downregulation of NMDA receptors leading to vagal dysregulation, and increased intracranial pressure due to inflammation from the encephalitis. In patients diagnosed with ANMDARE it is imperative to monitor them for dysrhythmias. In the event the patient has symptomatic bradycardia or prolonged pauses, a temporary transvenous pacemaker should be inserted. Treatment with tumor resection and immunotherapy may reverse the cardiac dysrhythmias seen with ANMDARE obviating the need for a permanent pacemaker. Our case highlights the need to consider ANMDARE as an etiology in patients presenting with encephalitis and cardiac dysrhythmias.

## Figures and Tables

**Figure 2A.**
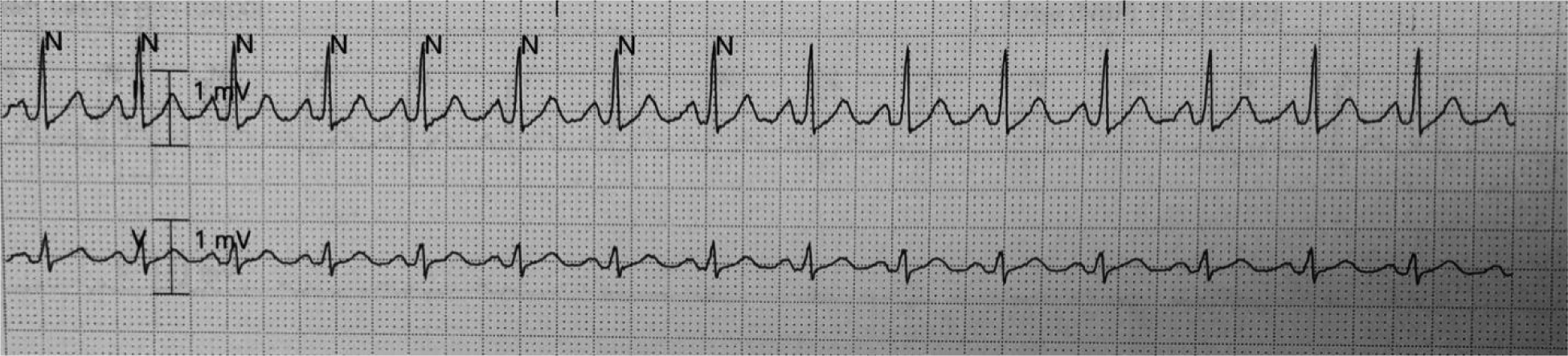
Sinus Tachycardia at 116 bpm

**Figure 2B.**
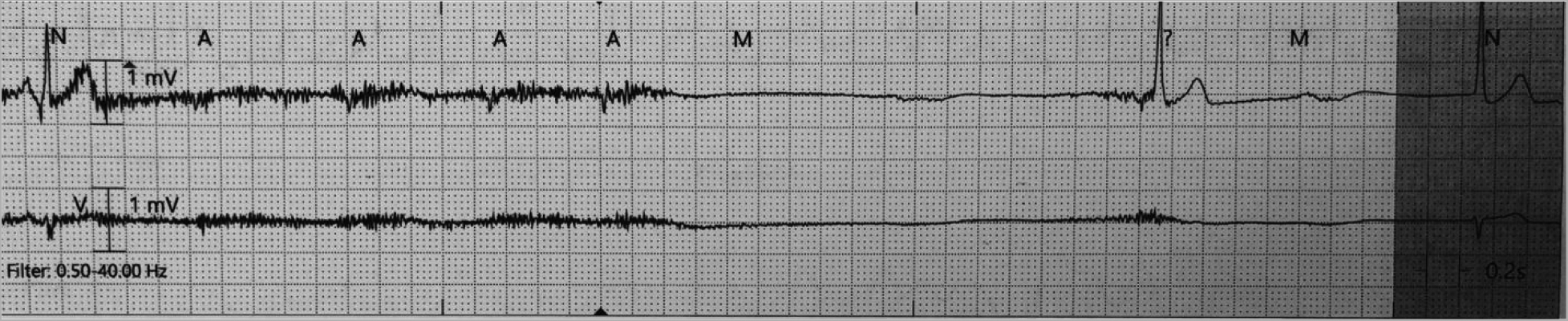
Sinus Pause of approximately 7 seconds followed by sinus bradycardia at a rate of 30 bpm

**Figure 4.**
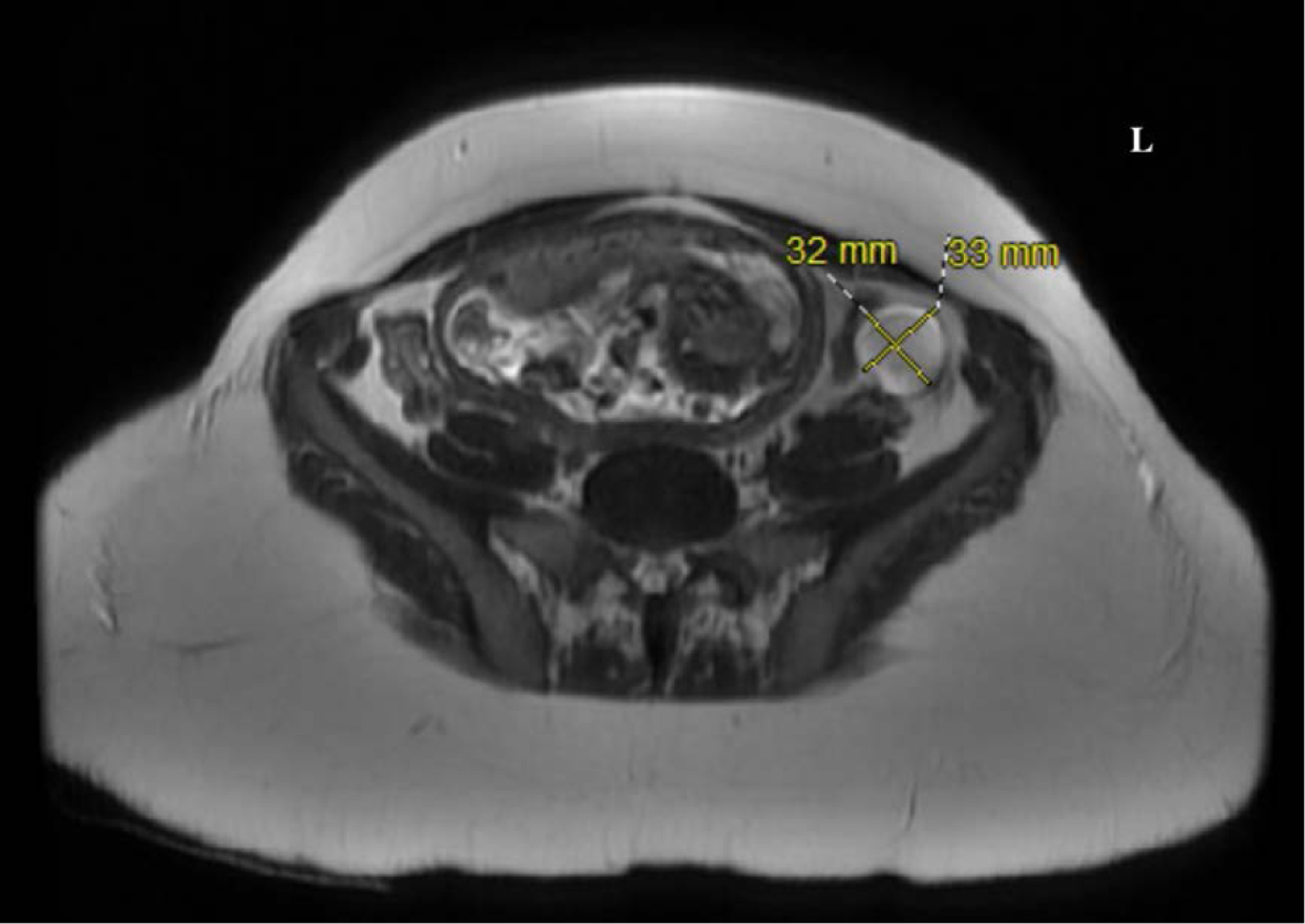
Pelvic MRI demonstrating intrauterine gestation and a left ovarian complex mass with layering debris approximately 33mm × 32mm in size

**Table 1.** Admission workup for altered mental status

**Comprehensive Panel**	
Na	141
K+	3.4
Cl−	104
C02	21
BUN	9
Cr	0.67
Glucose	101
Mg2+	2.24
P04	3.5
ALT	14
AST	12
ALP	57
**Microbiology**	
Parasite - Blood	Negative
SARS-CoV-2	Negative x3
Syphilis PCR	Negative
Blood Culture	Negative
Urine Culture	Negative
Sputum Culture	Negative
Hepatitis A/B/C/D/E	Negative
**Thyroid Function Test**	
TSH	1.28
Total T4	12
Free T4	1.16
**Arterial Blood Gas**	
Ph-Arterial	7.378
PC02-Arterial	36.2
PA02-Arterial	169 on 2L NC
Lactate	0.8
**Cerebral Spinal Fluid**	
Apearance	Clear
Total Cell Count	100
Glucose	71
Monocytes	2
WBC	64
RBC	44
Lymphocyte	98
Protein	39
Bacterial Culture	Negative
Mengitis Panel PCR	Negative
